# Telomeropathies in Interstitial Lung Disease and Lung Transplant Recipients

**DOI:** 10.3390/jcm14051496

**Published:** 2025-02-24

**Authors:** Brian D. Southern, Shruti K. Gadre

**Affiliations:** Integrated Hospital-Care Institute, Department of Pulmonary Medicine, Cleveland Clinic, Cleveland, OH 44195, USA; gadres@ccf.org

**Keywords:** telomere biology disorders, telomeropathies

## Abstract

Telomeropathies, or telomere biology disorders (TBDs), are syndromes that can cause a number of medical conditions, including interstitial lung disease (ILD), bone marrow failure, liver fibrosis, and other diseases. They occur due to genetic mutations to the telomerase complex enzymes that result in premature shortening of telomeres, the caps on the ends of cellular DNA that protect chromosome length during cell division, leading to early cell senescence and death. Idiopathic pulmonary fibrosis (IPF) is the most common manifestation of the telomere biology disorders, although it has been described in other interstitial lung diseases as well, such as rheumatoid arthritis-associated ILD and chronic hypersensitivity pneumonitis. Telomere-related mutations can be inherited or can occur sporadically. Identifying these patients and offering genetic counseling is important because telomerapathies have been associated with poorer outcomes including death, lung transplantation, hospitalization, and FVC decline. Additionally, treatment with immunosuppressants has been shown to be associated with worse outcomes. Currently, there is no specific treatment for TBD except to transplant the organ that is failing, although there are a number of promising treatment strategies currently under investigation. Shortened telomere length is routinely discovered in patients undergoing lung transplantation for IPF. Testing to detect early TBD in patients with suggestive signs or symptoms can allow for more comprehensive treatment and multidisciplinary care pre- and post-transplant. Patients with TBD undergoing lung transplantation have been reported to have both pulmonary and extrapulmonary complications at a higher frequency than other lung transplant recipients, such as graft-specific complications, increased infections, and complications related to immunosuppressive therapy.

## 1. Telomere Biology Disorders

Telomeres are regions of non-coding tandem repeat DNA sequences that are added to the end of all chromosomes to hinder progressive chromosomal shortening during replication of the cell. This prevents the chromosomes from becoming so short that they are no longer able to divide, which would result in cell death [1]. With aging, DNA replication does not always copy to the telomere end, so telomeres gradually become shorter, eventually causing senescence and cell death. Thus, in most somatic cells, telomere shortening is a consequence of aging. Not long after the discovery of telomeres, the existence of an enzyme with specific telomere terminal transferase-like properties that added repeat units to telomeric DNA to compensate for incomplete terminal replication was discovered [2]. Carol Greider, Elizabeth Blackburn, and Jack Szostak were awarded the 2009 Nobel prize in physiology or medicine for their work identifying the telomerase protein [3]. An oncologist and pediatrician in Dr. Grieder’s laboratory, Mary Armanios, was studying a population of mice that had been genetically modified to lack telomerase. Serendipitously, she was introduced by a colleague to a patient with aplastic anemia who was discovered to have a mutation in a gene called human telomerase reverse transcriptase, or hTERT, which provides critical instructions for making a component of telomerase. She discovered that, like her mice, this patient seemed to be becoming old “before his time”. She contacted his family members and collected blood for analysis. Three months later, the patient’s father died of liver disease and bone marrow failure at age 49. She discovered an aunt and uncle who both suffered from pulmonary fibrosis and would die in their 50s. All of these family members had the same abnormality in the TERT gene and suffered from a rare inherited disease called dyskeratosis congenita (DC) [4]. She went on to identify additional families who suffered from DC who had mutations in the gene DKC1, which also encodes for a component of telomerase, and who later developed idiopathic pulmonary fibrosis (IPF) [5].

**Figure 1:**
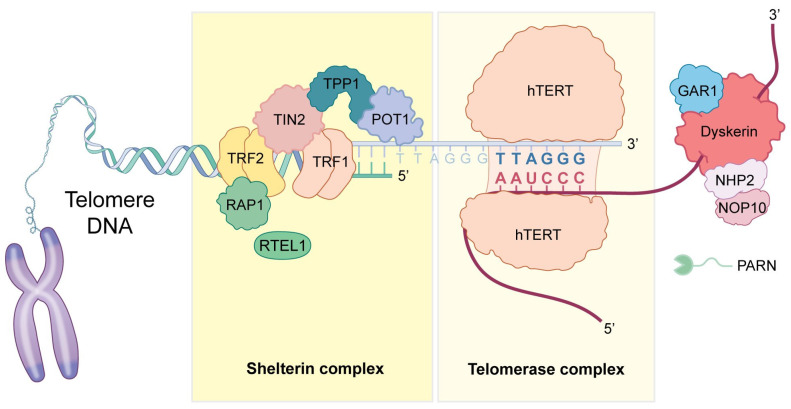
Telomerase and telomerase-associated proteins that are affected in telomeropathies associated with IPF and other ILDs.

It is now known that telomerase is composed of two key components, hTERT, the reverse transcriptase component of telomerase, and hTR (or TERC), the RNA portion that serves as the template for nucleotide addition by hTERT (Figure 1). Mutations in these genes result in short telomeres associated with aplastic anemia and pulmonary fibrosis, which can lead to premature death [6]. Other genes important in telomerase biology include TINF2 and other members of the shelterin complex, which caps, protects, and regulates telomeres [7]. The dyskerin complex is essential for regulating the accumulation of hTR and is therefore important in maintaining proper telomerase activity [8]. RTEL1 (DNA helicase in telomerase maintenance) regulates telomere elongation [9], and PARN (poly(A)-specific ribonuclease) is involved in 3′-end maturation of the telomerase RNA [10]. Mutations in any of these components can lead to shortened telomeres, causing IPF and other disorders [11,12,13,14] (Table 1).

In addition to IPF, shortened telomeres have been associated with bone marrow failure syndromes [30], emphysema [31], cryptogenic liver cirrhosis [32], premature hair graying [33], avascular necrosis of the hips and shoulders [34], periodontal disease [35], and increased predisposition to various types of myelodysplasia/malignancies [36]. Patients with any of these manifestations may be said to have a TBD, short telomere syndrome (STS), or a telomeropathy. All modes of genetic inheritance can be seen in the TBDs. DKC1 is the only X-linked telomere-related gene that can result in X-linked recessive disease. Four TBD-causing genes have been shown to have an autosomal recessive mode of inheritance, and carriers are asymptomatic, although they may manifest TBD-related disorders as adults. TINF2 variants are strictly autosomal dominant. The remaining eleven known TBD-related genes can be autosomal dominant or recessive and can be represented by early manifestation of disease [37]. Genetic anticipation can occur in TBD with individuals who carry autosomal dominant variants and pass the mutation, along with shortened telomeres, to their children [38]. This can result in an earlier onset of disease with each successive generation. This has been observed in families with pulmonary fibrosis carrying monoallelic variants of TERC, TERT, or other genes, resulting in early-onset pulmonary fibrosis and other disorders, such as bone marrow failure [39]. In addition, the phenomenon of phenocopy can occur, as shown in a systematic study of 99 families with familial pulmonary fibrosis, where five individuals lacked the TBD gene mutation but had shortened telomeres and developed pulmonary fibrosis [40]. The primary determinant of whether or not a TBD will develop is the presence of shortened telomeres, as the absence of telomerase by itself may not be sufficient to cause disease [41]. In mouse models with telomerase deletion, no phenotypic changes are observed, but phenotypic changes related to aging can be seen after several generations [42]. The true prevalence of TBDs is unknown, largely due to the incomplete penetration of disease features in many carriers. Patients with IPF, aplastic anemia, or cryptogenic cirrhosis may have a subtle clinical phenotype and may not be tested for telomeropathies.

Several methods have been developed to measure telomere length, and telomere restriction fragment length analysis is the gold standard. However, its utilization is limited as large amounts of DNA are needed for analysis [43]. Flow-FISH (fluorescence in situ hybridization) of blood involves the use of a fluorescent probe that labels telomeres and uses flow cytometry to analyze the telomere length in specific cell types, most commonly granulocytes and leukocytes. Flow-FISH takes into account the age-related telomere length, which is necessary because telomere attrition is a normal process in aging. The presence of a telomere length at or below the lowest 10th percentile of normal length is diagnostic for short telomere syndrome [44].

## 2. Telomere Disorders in IPF

IPF is the most prevalent interstitial lung disease (ILD), with an annual incidence rate of approximately 10 cases per 100,000 people in Europe and North America [45]. IPF is the most frequent manifestation of TBD in adults [6]. Mutations related to telomeres have been reported in approximately 25% of familial instances and 1–3% of sporadic IPF instances, a percentage that is greater than that of patients exhibiting hematologic phenotypes [46]. Genetic studies in IPF have revealed that around 40% of familial IPF cases and 25% of sporadic IPF cases exhibit shortened telomeres, suggesting hence-undiscovered gene mutations in IPF [47,48]. Importantly, there is evidence to support the notion that short telomere syndrome plays a greater role in sporadic IPF. First, it has been shown that patients with IPF, irrespective of whether there is a telomere mutation, have short telomeres compared to the population, with approximately half having a leukocyte telomere length (LTL) below the 10th age-adjusted percentile [49]. In fact, the severity of telomere shortening in sporadic IPF is like that seen in telomerase mutation carriers. Second, in a Mendelian randomization study that compared 82 other adult-onset diseases, pulmonary fibrosis had the strongest association with telomere length, with an estimated effect size of 10-fold [50]. Finally, it is clearly observed that patients with sporadic IPF and shortened telomeres can have the same extrapulmonary manifestations seen in those with familial IPF [48]. These lines of evidence support the premise that shortened telomeres are common in sporadic IPF, and telomere length, not necessarily the underlying mutation, has the greatest impact on the development of sporadic IPF. Thus, telomere shortening alone, without a mutation in a telomere-related gene, is a heritable trait that can occur and result in IPF. The most common pattern seen radiographically or histologically in patients with IPF and with or without shortened telomeres is the usual interstitial pneumonia pattern (UIP). However, it has been well established that patients with inheritable forms of IPF can present with “atypical” findings on imaging or pathology, and affected members with the same germline mutation may have discordant diagnoses. In an analysis of 15 families where at least two members were diagnosed with ILD through a multidisciplinary approach, it was found that 12 families (80%) had differing diagnoses, while only 3 families (20%) had a consistent diagnosis of IPF [51]. For example, in one family harboring the same TERC mutation, individual family members had histologic diagnoses that were consistent with UIP, chronic hypersensitivity pneumonitis, desquamative interstitial pneumonia, and pleuroparenchymal fibroelastosis [51].

There are currently no guidelines informing clinicians on how to proceed if genetic IPF is suspected. Offering genetic counseling and screening to all patients with IPF could be useful in counseling family members with shortened telomeres to avoid substances that are harmful to the lung, such as those associated with smoking or other inhaled exposures [52]. The average age of patients carrying a telomerase mutation is younger than that of those without mutations [53], and individuals with shorter telomeres have been found to experience faster disease progression and reduced survival rates without a transplant [51]; identifying these patients earlier could lead to closer monitoring and more aggressive treatment. Moreover, a subsequent analysis of patients from the PANTHER-IPF trial revealed that administering prednisone, N-aceylcysteine, and azathioprine to patients with a leukocyte telomere length below the 10% percentile of normal correlated with a higher combined outcome of death, lung transplantation, hospitalization, or a decrease in FVC [54]. These findings were replicated in additional cohorts. Avoidance of immunosuppressant therapy in patients with shortened telomeres could lead to improved outcomes. These examples demonstrate how a precision medicine approach using telomere length analysis could lead to changes in treatment strategies and patient care.

## 3. Telomere Disorders in Other ILDs

Over the past decade, telomere-related mutations and short telomeres have been implicated in various forms of non-IPF ILD. Examples include non-specific interstitial pneumonia (NSIP), pleuroparenchymal fibroelastosis (PPFE), hypersensitivity pneumonitis, and combined pulmonary fibrosis and emphysema [55]. Using whole-exome sequencing, it was demonstrated that TERT, RTEL1, and PARN mutations occur in patients with rheumatoid arthritis-associated ILD at a 3-fold higher odds ratio than they do in a control population. Furthermore, LTLs in patients with RA-ILD and TERT, RTEL1, or PARN mutations were shorter compared to those in control patients [56]. These data support the contribution of shortened telomeres to the risk of developing RA-ILD. In a prospective cohort study comparing chronic hypersensitivity pneumonitis patients on mycophenolate with shortened peripheral blood LTL and those with normal telomere length, every quartile reduction in LTL was linked to a similar decrease in survival without a transplant [57]. In patients with chronic hypersensitivity pneumonitis, an observational study of two independent cohorts showed an association between the extent of radiographic fibrosis and short telomere length and, similarly to previous studies in IPF, reduced survival in the short telomere population [58]. In a different study that contrasted patients with UIP against those with interstitial pneumonia with autoimmune features (IPAF) and connective tissue disease-associated interstitial lung disease (CTD-ILD), it was found that telomere length was greater in patients with idiopathic pneumonia with autoimmune features and CTD-ILD compared to those with UIP [59]. Among the IPAF patient group, those with LTL less than the 10th percentile had a more rapid decline in lung function and poorer transplant-free survival compared to those with LTL greater than the 10th percentile. RA-ILD patients had shorter LTLs than non-RA CT-ILD patients, but this was not associated with any differences in transplant-free survival. Telomere length in unclassifiable ILD was linked to transplant-free survival, even when potential confounding factors were taken into account. Patients whose telomere length was in the lowest quartile had almost the same transplant-free survival rate as IPF patients [60]. As seen in these various studies and others, a shortened LTL is usually associated with worse outcomes regardless of the ILD phenotype, perhaps more associated with the histological pattern than the clinical diagnosis.

## 4. Treatment of Telomere Biology Disorders in ILD

Currently, there is no available treatment for TBDs other than transplanting the organ that is failing. Based on a theory that there is an estrogen-responsive element in the TERT gene, the NIH conducted a phase 1–2 study where the synthetic sex hormone danazol was administered to patients with various manifestations of TBD, with the primary outcome being a 20% decrease in the annual rate of telomere attrition at 24 months [61]. The study was halted prematurely as 12 of the patients met the primary endpoint. In almost all of these patients (11/12), there was an increase in telomere length at 24 months compared to the baseline length, and there was an improvement in blood counts. Based on this work, danazol was administered to 50 patients with IPF after they showed progressive disease with either of the two approved therapies for IPF, pirfenidone or nintedanib, with the primary outcome being a change in forced vital capacity (FVC) over 12 months of treatment [62]. After one year, only 22% (11 patients) continued the danazol treatment, while 39 patients had ceased therapy, predominantly due to side effects (56%) or death (33%). The lung function of all patients significantly deteriorated, with an average FVC reduction of 485 mL observed in the year following the commencement of treatment. Despite this negative study, other studies utilizing danazol are currently ongoing. High-throughput screening has identified an inhibitor of PAPD5 (a non-canonical polymerase that oligoadenylates and destabilizes TERC) called BCH001 that restores telomere length in induced pluripotent stem cells from patients with DC [63]. Imetelstat, a lipid-conjugated oligonucleotide that competitively blocks telomerase, was examined in a preliminary myelofibrosis study, and 21% of the participants achieved either partial or full remission [64]. A separate trial involving imetelstat in patients with advanced non-small cell lung cancer and shortened telomeres indicated a potential trend of improvement in both progression-free and overall survival [65]. The progression of fibrosis in a mouse model with short telomeres exposed to bleomycin was halted by activating telomerase using an adenovirus-associated vector (AAV) [66]. These investigators went on to show that physiologic aging leads to short telomeres and fibrosis in the lung of mice, and that administration of their AAV gene therapy prevented the onset of fibrosis [67]. These and other exciting strategies to reverse telomere shortening are novel and may be potential future therapeutic options for patients with TBD.

There are currently two approved therapies for IPF (pirfenidone, nintedanib) [68,69] and one approved therapy for progressive pulmonary fibrosis (nintedanib) [70]. There have been studies demonstrating that the use of these anti-fibrotic therapies in patients with telomerase gene mutations is safe and efficacious in slowing FVC decline [71]. A post hoc analysis of the pirfenidone trials CAPACITY and ASCEND revealed a significant treatment advantage for patients with telomere lengths both above and below the median. Nevertheless, individuals with telomere mutations underwent a more rapid decrease in FVC compared to those without mutations [53]. A retrospective analysis showed that both pirfenidone and nintedanib were linked to a slower rate of FVC decline compared to the rate prior to treatment [72]. These studies support the safety of using anti-fibrotics in progressive fibrosis regardless of telomere length.

## 5. Telomere Biology Disorders and Lung Transplantation

The only intervention that can extend the life of patients with end-stage pulmonary fibrosis is lung transplantation. Among patients with ILD who are referred for lung transplantation, it has been discovered that up to 25% possess a genetic variant associated with TBDs [73]. This percentage is believed to be higher due to the typically earlier onset and increased severity of ILD associated with TBD. The prevalence of TBDs is reported to be two times higher in IPF patients who receive a lung transplant compared to those with IPF who do not have a transplant [73]. In a particular group, it was found that 32% of IPF lung transplant recipients had a telomere length that was less than the 10th percentile [74].

Patients with TBD undergoing lung transplantation have been reported to have both pulmonary and extrapulmonary complications at a higher frequency than other lung transplant recipients. These include airway complications such as anastomotic dehiscence or bronchial stenosis [75], chronic allograft dysfunction [75], and severe infectious, hematological, hepatic, renal, and gastrointestinal complications [75,76,77,78]. The identification of telomere biology disorders may allow for strategies to prevent complications of short telomere syndrome in lung transplant recipients. It should be noted that the presence of a telomere biology disorder by itself is not considered to be a contraindication to lung transplantation.

Standard post-lung-transplant immunosuppressive protocols include a combination of calcineurin inhibitors (tacrolimus/cyclosporine), targeting varying trough levels, steroids (prednisone/dexamethasone), and antimetabolites (mycophenolate mofetil/azathioprine). Standard infection prophylaxis protocols include lifelong pneumocystis jirovecii pneumonia (PJP) prophylaxis (trimethoprim–sulfamethoxazole/pentamidine/dapsone) and varying durations of cytomegalovirus (CMV) (valganciclovir) and fungal (posaconazole/itraconazole/voriconazole) prophylaxis depending on the transplant center. These protocols often need to be modified in TBD patients to improve medication tolerance and mitigate potential adverse outcomes.

## 6. Graft-Specific Complications

Research comparing lung transplant recipients with telomere biology disorders to recipients with normal telomere length has produced inconsistent results regarding survival and graft outcomes such as Primary Graft Dysfunction (PGD), acute cellular rejection (ACR), and Chronic Lung Allograft Dysfunction (CLAD). In a single-center observational cohort of 82 patients with IPF, Newton and colleagues reported that short telomere length was associated with increased rates of PGD and CLAD [74]. There was nearly an 11-fold increased risk of death in the short telomere cohort compared to recipients with normal telomere length. Courtwright et al. reported that a shorter telomere length was linked to reduced CLAD-free survival [79]. In contrast, a more recent study by Alder et al. showed that post-transplant survival and CLAD-free survival did not differ with the telomere length within IPF lung transplant recipients [73]. This inconsistency in results could potentially be due to the fact that the definition of short telomeres was not uniform across different studies.

Acute cellular rejection is a less frequent occurrence in lung transplant recipients with TBD compared to recipients with normal telomere length. This is predominantly attributed to T-cell immunosenescence in patients with TBD. In Snyder et al.’s research, it was discovered that IPF lung transplant recipients with short telomeres experienced premature “aging” in their circulating T-cell compartment and a significant age-related decrease in early ACR burden [80]. While lung transplant recipients with telomere biology disorders may exhibit lower rates of acute graft rejection, they remain susceptible to chronic rejection.

## 7. Wound Healing and Airway Issues

The literature on wound healing and airway issues in lung transplant recipients with shortened telomeres is sparse. In a retrospective multicenter study of 63 lung transplant recipients with a telomere length in the <10th percentile, increased rates of dehiscence or stenosis in the large airway were reported in the short telomere group compared to a Scientific Registry of Transplant Recipients (SRTR) control group [81]. Individuals who have received a lung transplant and have TBD are more susceptible to complications in healing their airways. This is due to the premature aging of various cells such as fibroblasts, immune cells, endothelial cells, and epithelial cells. Infectious complications further complicate airway healing issues. The procedure of direct bronchial artery revascularization (BAR) during lung transplant surgery has been reported to lessen airway ischemia and enhance the healing of the airway in the early postoperative phase [82,83]. The feasibility of BAR at the time of transplant surgery should be considered in potential lung transplant recipients with TBD at the time of listing. Expert opinion also suggests that lung transplant recipients with TBD are at increased risk for sternal and chest wall wound dehiscence at a higher rate compared to recipients with normal telomere length, although the published literature is lacking.

## 8. Hematological Complications

Patients with TBD are more likely to develop a reduction in blood cells when given medications that suppress bone marrow, which are usually well tolerated in individuals without short telomeres. Silhan et al. compiled an international series of individuals carrying telomerase mutations who underwent lung transplantation in the USA, Australia, and Sweden [78]. Seven recipients were included in the study, with a median follow-up of 1.9 years. The predominant complications in this group were of a hematological nature, with 88% of recipients needing platelet transfusion assistance. All recipients required an adjustment in their immunosuppressive medications. Most patients were able to tolerate only two drug immunosuppressive regimens. The platelet counts increased after the immunosuppressive drugs were modified; however, they stayed below the levels before the transplant.

Short telomere length is a risk factor for clinically significant leucopenia as well [78]. In another study involving IPF lung transplant recipients with TBD, it was more common for patients with short telomeres to have their immunosuppression medications stopped due to cytopenias, compared to recipients without IPF. Bone marrow dysfunction requiring a bone marrow biopsy and increased requirements for transfusions and growth factor support were also noted among short telomere patients [84]. These studies emphasize the delicate state of the bone marrow reserve in patients with short telomeres and lung disease, which may become apparent after a transplant.

Strategies to prevent bone marrow suppression involve modifying the dosage of standard immunosuppression treatments by either pausing or reducing the amount of antimetabolite therapy, altering CMV prophylaxis, or using dapsone in place of trimethoprim/sulphamethoxazole for PJP prophylaxis [78]. The additional treatment for post-transplant cytopenias is mostly supportive. The use of blood or platelet transfusions and granulocyte colony-stimulating factor for neutropenia has been implemented. Danazol is typically not used in this patient group due to insufficient evidence and the potential danger of developing myelodysplastic syndrome (MDS) and acute myeloid leukemia (AML) [85]. In some instances, due to the heightened risk of MDS and AML, a bone marrow biopsy may be required to investigate cytopenias [84]. It is beneficial to include hematologists in the multidisciplinary team caring for lung transplant recipients with TBD.

## 9. Immunity and Infectious Complications

The decrease in T-cell numbers and function with age is referred to as T-cell immunosenescence. In patients with TBD, T cells show premature aging phenotypes compared to those in patients with normal telomere length. Short telomere syndromes in children and adults, caused by apoptosis induced by short telomeres and secondary depletion of T-cell precursors, result in low T-cell counts. These syndromes also lead to significant functional impairments, such as depleted pools of naïve T cells and a limited T-cell repertoire [79]. In a study group of 26 patients with TBD at Johns Hopkins, all of whom were under 60 years old, 32% (or 9 individuals) developed opportunistic infections linked to T-cell immunodeficiency. The infections were mainly related to herpes viruses (cytomegalovirus and varicella zoster virus), accounting for 66% of the infections. When CMV led to end-organ disease, such as encephalitis or pneumonitis, the infection resulted in death. Interestingly, adults can develop immunodeficiency without bone marrow failure, which can make them susceptible to life-threatening opportunistic infections.

An increased risk for other infectious complications is seen in lung transplant recipients with TBD as well. In an international case series of lung transplant recipients with TBD, 85% of the patients developed infectious complications with the first 2 years after transplantation [78]. The primary infections reported were Gram-negative pneumonia/sepsis and opportunistic infections, like pulmonary aspergillosis and cytomegalovirus pneumonitis.

Similarly, lung transplant recipients with TBD remain at higher risk for herpes viridae infections due to T-cell immunodeficiency [86]. Recipients of lung transplants with IPF and TBD have a risk that is up to five times higher for recurring CMV viremia [77]. These patients are also at higher risk of CMV end-organ disease compared to age-matched controls with and without IPF [77]. Simultaneously, multiple factors can make the treatment of CMV viremia more complex in this group. Lung transplant recipients with TBD are prone to CMV drug resistance and may experience bone marrow suppression as a side effect of antiviral drugs such as ganciclovir and valganciclovir. The use of alternative treatments such as foscarnet or cidofovir can be restricted in some circumstances due to their side effects like kidney and bone marrow suppression and poor tolerance. Letermovir, a new anti-CMV medication, targets the viral terminase complex, which is involved in the latter stages of the viral replication process. It has received approval for preventing CMV infection in allogenic stem cell transplants. Letermovir was examined for off-label use in preventing CMV in thoracic transplant patients, including 37 individuals who had lung transplants, and it was found to be both safe and successful [87]. Letermovir was used as primary prophylaxis in 62% and secondary prophylaxis in 38% of the patients due to myelosuppression or prior CMV resistance. Adverse effects such as myelosuppression, nausea, and lightheadedness required the cessation of letermovir in 12% of the patients. There were 12 episodes of low-level CMV DNAemia that did not require letermovir cessation and only 1 episode of clinically significant breakthrough CMV infection [87]. Additional research is needed to ascertain whether letermovir could be effective for CMV prevention, particularly in lung transplant recipients with TBD.

Iasella et al. found that lung transplant recipients with IPF were at a higher risk of developing post-transplant lymphoproliferative disorder associated with the Epstein–Barr virus (EBV), which they hypothesized was due to immune dysfunction related to short telomeres [88].

When designing care protocols to manage lung transplant recipients with TBD, consultation with transplant infectious disease physicians to consider alternative agents to prevent and manage infections is imperative. Lifelong CMV/HSV and antifungal prophylaxis and prolonged durations of antimicrobial therapy for other infections may be considered.

## 10. Liver Disease and Cirrhosis

Approximately 10% of patients with short telomeres have been reported to have liver abnormalities. Severe short telomere syndrome is typically associated with a classic triad of IPF, liver disease, and bone marrow failure [88]. Several case studies have reported successful early outcomes for patients with TBD who have undergone combined liver and lung transplants due to end-stage IPF and cirrhosis [89,90]. It is advised to conduct early screening for liver dysfunction in patients identified with short telomere length before the transplant period. Continued surveillance for liver disease with appropriate laboratory evaluation, imaging, and hepatology evaluation should be carried out in the post-transplant period.

## 11. Other Complications

Lung transplant recipients with TBD have been described to have a higher incidence of renal dysfunction after transplantation. Tokman et al. described outcomes in 14 lung transplant recipients with telomerase mutations, of which more than half developed acute renal failure, and 83% of the recipients developed chronic renal insufficiency during 3 years of follow-up [75]. Kidney biopsies from patients needing extended dialysis revealed tubular abnormalities and epithelial cell vacuolization, which are strongly indicative of toxicity from calcineurin inhibitors [78]. These findings suggest that lung transplant recipients with TBD may be more susceptible to the nephrotoxic effects of calcineurin inhibitors. It is plausible that patients with subclinical liver disease are prone to reduced clearance, as calcineurin inhibitors are metabolized by the hepatic p450 enzyme.

Gastrointestinal bleeding requiring blood transfusions was reported in more than one-third of lung transplant recipients with TBD in the cohort described by Silhan and colleagues [78]. Interestingly, in one of the patients, the bleeding was secondary to ischemic colitis with histopathologic findings documented in telomere-related enteropathy [78].

## 12. Comprehensive Care of Lung Transplant Recipients with Telomere Biology Disorders

Before transplantation, it might be justified to establish a diagnosis of underlying TBD due to the frequency of TBD in lung transplant recipients, potential post-transplant complications, and the need to alter post-transplant protocols. Recognizing short telomeres may enable the development of methods to avoid permanent complications of short telomere syndrome in patients who have undergone lung transplants. Table 2 and Table 3 provide a suggested multidisciplinary team evaluation of STS in the pre- and post-transplant period. A multidisciplinary approach will help facilitate early diagnosis with screening protocols, early genetic counselling, and risk stratification and mitigation strategies such as induction protocols, modified immunosuppression protocols, and assessments for dual organ transplantation.

## 13. Conclusions

Telomeres and their associated proteins are essential for maintaining the telomere length in dividing cells, preventing cellular senescence and cell death. A number of mutations in the components of the telomerase complex, the shelterin complex, and other associated proteins have been implicated in the development of IPF and other ILDs. Approximately 25% of familial cases and 1–3% of sporadic IPF cases have been found to have telomere-related mutations. They have also been described in cases of ILD, such as in rheumatoid arthritis and hypersensitivity pneumonitis. Currently there is no available treatment for TBDs other than transplanting the affected organ, although studies are ongoing using molecules that lengthen the telomere or enhance telomerase activity. There has been an increasing interest in the impact of telomere length in lung transplant recipients. Telomere length could potentially be used as a pre-transplant biomarker to evaluate the risk of outcomes after transplantation. Nonetheless, one should be cautious when interpreting the limited and inconsistent evidence from a series of studies conducted at a single center. It is not adequate to disqualify lung transplant recipients from listing solely based on the length of their telomeres. There remain significant unknowns regarding standardizing the approach to care in this patient population. It is clear, however, that the complexity of these patients necessitates a comprehensive multidisciplinary approach to the pre- and post-lung-transplant care of this patient population.

## Figures and Tables

**Table 1:** Known mutations in telomerase and shelterin complex subunits.

Gene	Chromosome	Inheritance	Effect of Mutation on Telomerase Complex	Refs.
DKC1	Xq28	XLR	Decreases TERT/TERC expression, suppresses telomerase activity	[15,16]
TERC	3q26.3	AD	Slows or stops cell proliferation independent of telomerase activity, accelerates shortening of longer telomeres, increases oxidative DNA damage at telomeres	[17,18,19]
TERT	5p15.53	AD, AR	Loss of function of the telomerase complex	[11,20]
NOP10	15q14-q15	AR	Diminishes telomerase activity via TERC downregulation	[21]
NHP2	5q35.5	AR	Diminishes telomerase activity via TERC downregulation	[22]
TINF2	14q11.2	AD	Lack of stabilization of TRF1 on telomeres; loss of protection of telomere and genome stability	[23,24]
RTEL	20q13.33	AR	Lack of disassembly of DNA loops for proper access to the telomere; lack of telomere stability	[25,26,27]
PARN	16p13.12	AD, AR, XLR	Does not allow for removal of poly(A) tails from hTR, causing lack of maturation of telomerase	[28,29]

XLR = X-linked recessive, AD = autosomal dominant, AR = autosomal recessive.

**Table 2:** Comprehensive pre-transplant care of lung transplant recipients with telomere biology disorders.

Consult to hematology to evaluate cytopenias and need for bone marrow biopsy
Consult to genetic counselling for assessment of familial risk of telomere biology disorder
Consult to hepatology for evaluation of liver dysfunction, appropriate imaging, and need for liver transplantation
Consult to infectious disease for counselling regarding infectious risks and to determine perioperative antibiotic choice and duration
Determine surgical feasibility of bronchial artery revascularization at time of lung transplant surgery to decrease risk of airway complications
If lung transplant candidate with telomere biology disorder is CMV IgG negative and stable, it would be ideal to consider CMV-negative donor, if possible

**Table 3:** Comprehensive post-transplant care of lung transplant recipients with telomere biology disorders.

Immunosuppression modification: avoid antimetabolite use, early reduction in steroid dose
Early initiation of antifungal and antiviral prophylaxis
Seek advice from infectious disease specialist for handling antimicrobial and antiviral prophylaxis in context of cytopenias
Consult to hematology to evaluate cytopenias and need for bone marrow biopsy
Consult to interventional bronchoscopy for expeditious management of airway complications
Consult to hepatology for continued surveillance of liver dysfunction
Age-appropriate cancer screening and yearly dermatology evaluation for skin cancer assessment
